# Data on the association of CMPK1 with clinicopathological features and biological effect in human epithelial ovarian cancer

**DOI:** 10.1016/j.dib.2017.05.022

**Published:** 2017-05-12

**Authors:** Daibing Zhou, Lingyun Zhang, Qunbo Lin, Weimin Ren, Guoxiong Xu

**Affiliations:** a Center Laboratory, Jinshan Hospital, Fudan University, Shanghai 201508, China; b Department of Oncology, Shanghai Medical College, Fudan University, Shanghai 200032, China

**Keywords:** TGF-β signaling, UMP/CMP kinase, Tissue microarray, Tumorigenesis, miRNA, Therapeutic target

## Abstract

Human epithelial ovarian cancer (EOC) is the most lethal gynecological disease. However, the molecular mechanisms by which transforming growth factor-β (TGF-β) regulates ovarian tumor progression markers remain unclear. The present data show cytidine monophosphate kinase (CMPK) as an EOC biomarker and are related to the article entitled “Cytidine monophosphate kinase is inhibited by the TGF-β signalling pathway through the upregulation of miR-130b-3p in human epithelial ovarian cancer” [1]. CMPK, as well as cystatin B [2] and β-2-microglobulin [3], is overexpressed in human epithelial-type ovarian tumors. CMPK is an enzyme required for nucleic acid biosynthesis [4] and is regulated by the TGF-β signaling pathway in EOC cells [1]. Furthermore, the data show the effect of CMPK-shRNA on EOC cell apoptosis and TGF-β-induced Smad2 phosphorylation. CMPK expression in two EOC cell lines OVCAR-3 and SK-OV-3 is regulated by multiple miRNAs and some of these miRNAs may affect EOC chemoresistance [5].

**Specifications Table**

**Table:** 

Subject area	*Cell biology; Health science*
More specific subject area	*Apoptosis; Ovarian cancer*
Type of data	*Table and figure*
How data was acquired	*Tissue microarray, human EOC cell lines OVCAR-3 and SK-OV-3 (ATCC, Manassas, VA, USA), Transfection, Western blot, Flow cytometry (Becton Dickinson Beckman Coulter, Inc., Brea, CA, USA)*
Data format	*Analyzed*
Experimental factors	*Cells were transfected with siRNA or shRNA; Cells were treated with 10* *ng/ml TGF-β1 for 24 h*
Experimental features	*The tissue microarray included 100 paraffin-embedded ovarian tissues; Screen 9 miRNAs that potentially target CMPK1*
Data source location	*Shanghai, China*
Data accessibility	*The data are with this article*
Related research article	*Zhou et al.**[1]**“Cytidine monophosphate kinase is inhibited by the TGF-β signalling pathway through the upregulation of miR-130b-3p in human epithelial ovarian cancer” j.cellsig 35:197–207.*

**Value of the data**

• Data present CMPK as an ovarian serous tumor progression marker.
• The location of CMPK protein expression in the cytoplasm and nucleus of epithelial-type ovarian tumor cells is shown.
• Suppression of CMPK affects the doubling time of EOC cells.
• Data describe for the first time that knockdown of CMPK influences EOC cell apoptosis.
• Data show the effect of CMPK-shRNA on TGF-β-induced Smad2 phosphorylation.

## 1 Data

**Fig. 1:**
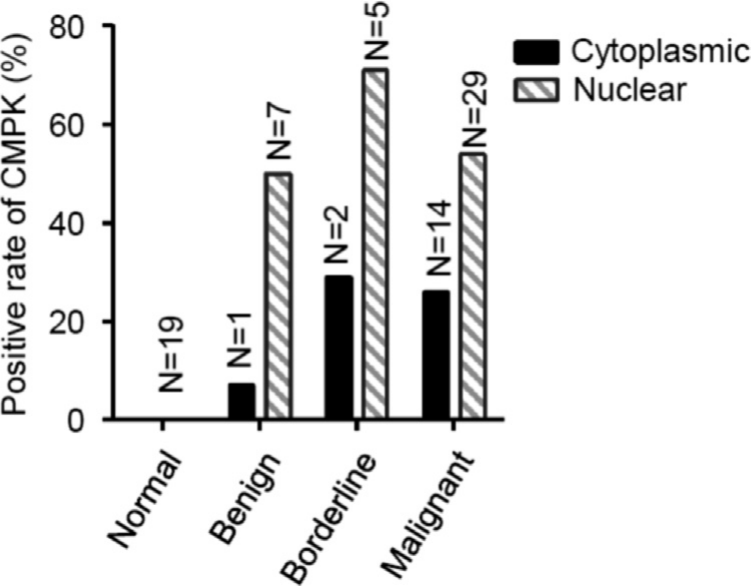
Evaluation of positive rate for CMPK staining in tissue microassay. CMPK protein staining in subcellular localization was scored. Histogram shows the positive rate of CMPK staining in different tissues. A positive staining of CMPK was found in the cytoplasm and nucleus of ovarian tumor cells, whereas normal tissue appeared negative staining. Data are presented as mean. N, case number; Normal, normal ovarian tissue; Benign, benign tumor; Borderline, borderline tumor; Malignant, malignant tumor.

**Fig. 2:**
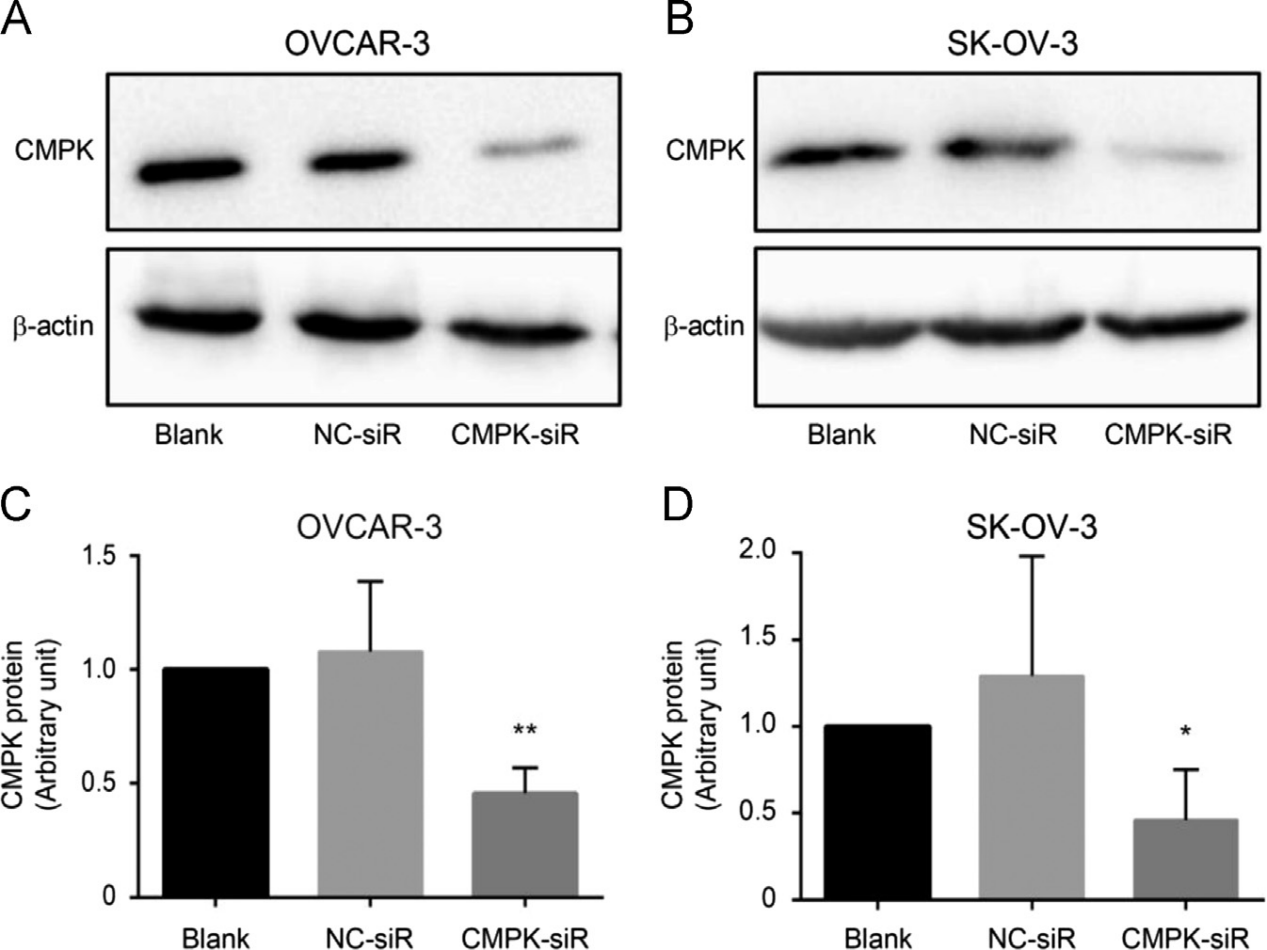
Knockdown of CMPK expression in EOC cells is confirmed by Western blot after siRNA transfection. OVCAR-3 (A, C) and SK-OV-3 (B, D) cells were transiently transfected with CMPK-siRNA (CMPK-siR) and negative control-siRNA (NC-siR), respectively. Untreated cells were used as blank control (Blank). (A, B) CMPK protein was detected by Western blot at 24 h post-transfection. (C, D) Histograms show semi-quantitative analysis after densitometry on the gels of (A) and (B), respectively. Data are presented as mean ± SEM (N = 3). *P<0.05; **P<0.01 (CMPK-siRNA *vs.* Blank or NC-siR).

**Fig. 3:**
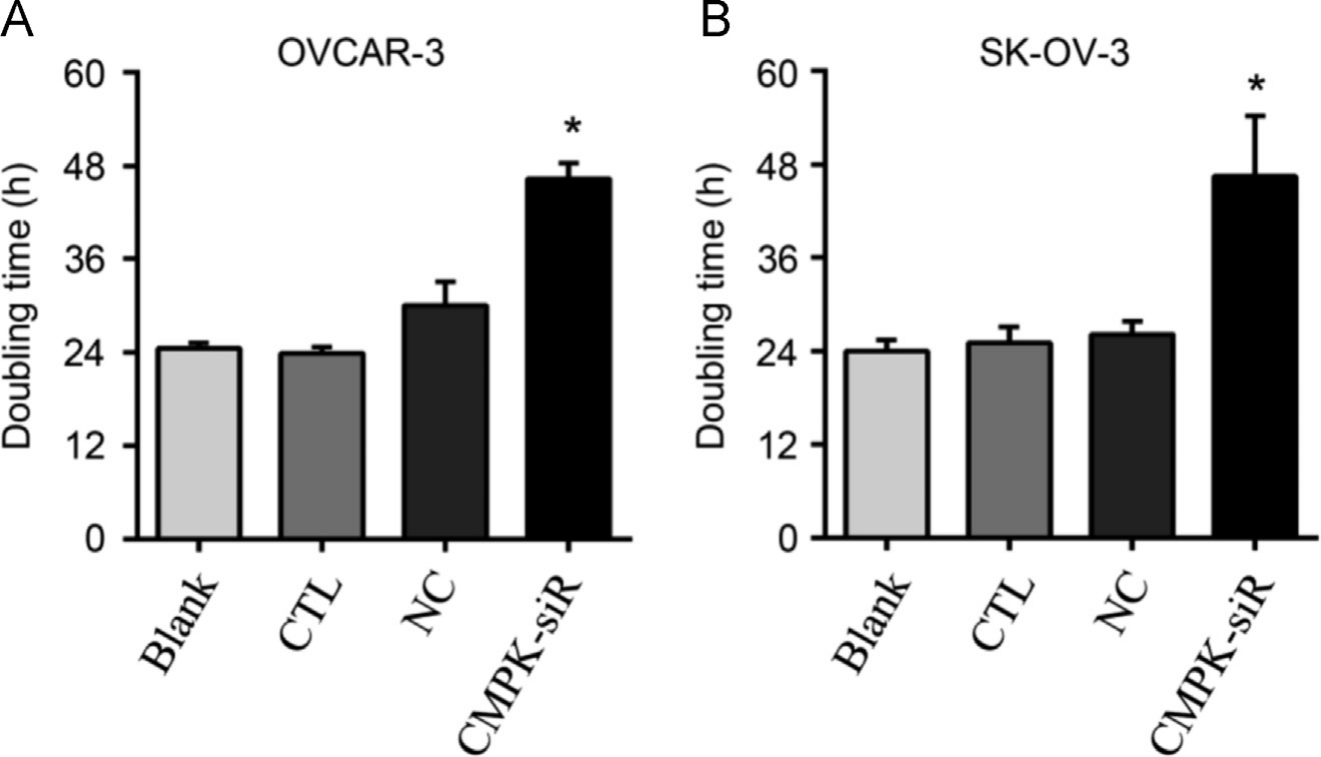
Doubling times (DT) based on the OD values at the time of measurement. (A) Histogram shows the DT (h, hour) of OVCAR-3 cells: Blank, 24.52±0.74; CTL, 23.91±0.82; NC, 30.07±3.02; CMPK-siR, 46.32±2.03. (B) Histogram shows the DT of SK-OV-3: Blank, 24.02±1.41; CTL, 25.06±2.06; NC, 26.19±1.64; CMPK-siR, 46.46±7.80. *P<0.05 (CMPK-siRNA group *vs.* other groups). Blank, control without transfection; CTL, reagent control, NC, negative control of transfection; CMPK-siR, CMPK-siRNA transfection.

**Fig. 4:**
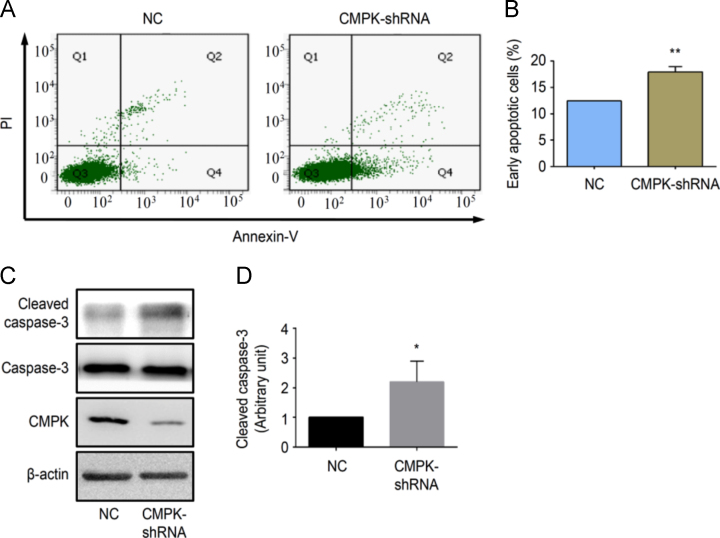
Suppression of CMPK influences EOC cell apoptosis. OVCAR-3 cells were infected with CMPK-shRNA lentiviral particles or empty vector lentiviral particles. (A) The proportion of apoptotic cells of OVCAR-3 CMPK-shRNA expressing cells (CMPK-shRNA) and negative control cells (NC) was determined by flow cytometry. (B) Histogram shows the quantitative analysis of the percentage of early apoptotic cells from (A). (C) Cleaved caspase-3 and full sized caspase-3 proteins in NC and CMPK-shRNA cells were detected by Western blot. (D) Histogram shows the semi-quantitative analyses of the gels from (C) after densitometry (N = 3). Data are presented as mean±SEM. *P<0.05; **P<0.01 (CMPK-shRNA *vs.* NC).

**Fig. 5:**
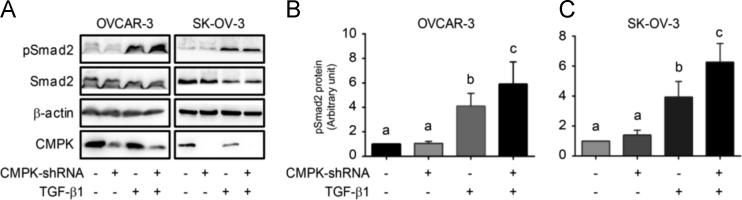
Effect of CMPK-shRNA on TGF-β-induced Smad2 phosphorylation. (A) Expression of protein detected by Western blot. (B, C) Histogram shows the semi-quantitative analyses of pSmad2 in OVCAR-3 and SK-OV-3 cells after densitometry in the gels from (A). Data are presented as mean±SEM. Different superscripts denote a significant difference from each other (P<0.05; N=3).

**Fig. 6:**
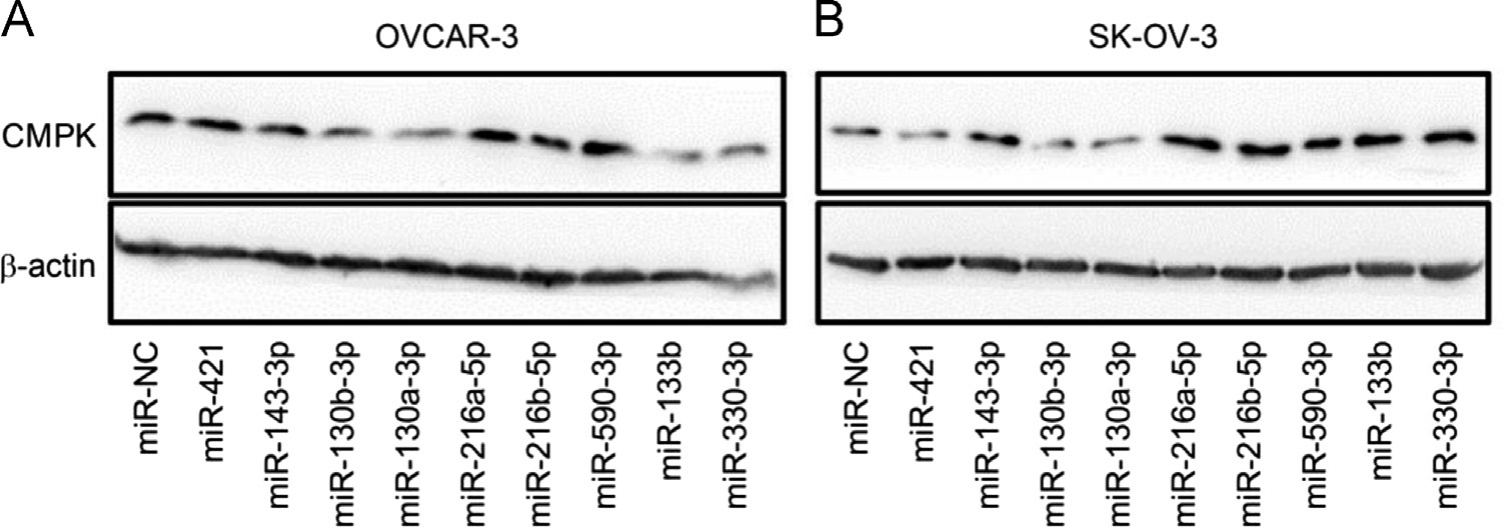
Effect of miRNAs on CMPK expression. CMPK protein expression was detected by Western blot. OVCAR-3 and SK-OV-3 cells were transiently transfected with nine miRNA mimics. Negative control miRNA (miR-NC) was used as control. Experiment was repeated twice.

The data represent the observation from experiments of tissue microarray, Western blot and flow cytometry. Data in Table 1 are the list of sequences of siRNA, shRNA, miRNA and PCR primer used in a related research article [1]. The data of the association of CMPK protein expression with clinicopathological features of patients with epithelial ovarian tumours are shown in Table 2. Data in Fig. 1 show the positive rate for CMPK staining in the cytoplasm and nucleus. Fig. 2 confirms the knockdown of CMPK protein by Western blot after CMPK-siRNA transfection in OVCAR-3 and SK-OV-3 cells. Doubling times (DT) based on the optical density (OD) values at the time of measurement is shown in Fig. 3. Data in Fig. 4 represent the proportion of early apoptotic cells detected by flow cytometry and the expression of cleaved caspase-3 protein, an active form of apoptotic protein, detected by Western blot after CMPK-shRNA infection. Data of phospho-Smad2 detection by Western blot are shown in Fig. 5. Screening data of the effect of miRNAs on CMPK expression are shown in Fig. 6.

**Table 1:** Sequences of siRNA, shRNA, miRNA and PCR primer.

Name	Sequence (5’ → 3’)	Target position
CMPK-siRNA		
Sense	GAAAGAUUGUACCAGUUGAtt	nt 424–442
Antisense	UCAACUGGUACAAUCUUUCtt	
NC-siRNA		
Sense	UUCUCCGAACGUGUCACGUTT	Scramble
Antisense	ACGUGACACGUUCGGAGAATT	
CMPK-shRNA		
Sense	gatccGAAAGATTGTACCAGTTGAttcaagagaTCAACTGGTACAATCTTTCttttttg	nt 424–442
Antisense	aattcaaaaaaGAAAGATTGTACCAGTTGAtctcttgaaTCAACTGGTACAATCTTTCg	
miR-130b-3p		
miR-130b mimic	CAGUGCAAUGAUGAAAGGGCAU	nt 1824–1834
miR-Ctrl	UCACAACCUCCUAGAAAGAGUAGA	
anti-miR-130b	AUGCCCUUUCAUCAUUGCACUG	nt 1824–1834
anti-Ctrl	UCUACUCUUUCUAGGAGGUUGUGA	
CMPK PCR primer		
Forward	TGTCAGCTCCCTCAGCGTC	nt 114–132
Reverse	CGAGGACGAACACGACCAG	nt 255–273
β-actin PCR primer		
Forward	CATTGCCGACAGGATGCAG	nt 1008–1026
Reverse	CTCGTCATACTCCTGCTTGCTG	nt 1155–1176
miR-130b-3p primer		
Forward	GGCAGTGCAATGATGAAAGG	
Reverse	GTGCAGGGTCCGAGGT	
U6 primer		
Forward	CTCGCTTCGGCAGCACA	
Reverse	AACGCTTCACGAATTTGCGT	
miR-130b RT primer	GTCGTATCCAGTGCAGGGTCCGAGGTATTCGCACTGGATACGACatgccc	

The target position in CMPK mRNA sequence (GenBank Accession: NM_016308) and β-actin mRNA sequence (GenBank Accession: NM_001101) is shown. Sequence in lowercase indicates a linker. NC, negative control; nt, nucleotide; PCR, polymerase chain reaction; siRNA, small interfering RNA; shRNA, short hairpin RNA.

**Table 2:** Association of CMPK protein expression with clinicopathological features of patients with epithelial ovarian tumours.

Clinicopathological features	n	Total CMPK expression		P-value
		Positive (%)	Negative (%)	
Age				0.221^a^
≤45	26	11 (42.31)	15 (57.69)	
>45	49	28 (57.14)	21 (42.86)	
Histological type				
Serous tumour	44			0.005^b^
Benign	5	0 (00.00)	5 (100.00)	
Borderline	5	5 (100.00)	0 (00.00)	
Malignant	34	14 (41.18)	20 (58.82)	
Mucinous tumour	14			0.748^b^
Benign	9	3 (33.33)	6 (66.67)	
Borderline	2	1 (50.00)	1 (50.00)	
Malignant	3	2 (66.67)	1 (33.33)	
Endometrioid adenocarcinoma	10	8 (80.00)	2 (20.00)	
Transitional cell carcinoma	4	4 (100.00)	0 (00.00)	
Adenocarcinoma	3	2 (66.67)	1 (33.33)	
FIGO stage				0.778^b^
I	24	13 (54.17)	11 (45.83)	
II	10	6 (60.00)	4 (40.00)	
III	19	10 (52.62)	9 (47.37)	
IV	1	1 (100.00)	0 (00.00)	

The expression of CMPK was detected by immunohistochemistry using tissue microarray. For comparison of CMPK expression associated with age, a χ^2^ test was applied

a For multiple comparisons of CMPK expression associated with and histological types and clinical stages, a Fisher׳s exact test was applied.

b n, number of cases; Positive, positive expression; Negative, negative expression; FIGO, International Federation of Gynaecological Oncologists. Normal ovarian tissues (n=19) showing a negation staining of CMPK are served as control.

## 2 Experimental design, materials and methods

### 2.1 Tissue microarray

Ovarian tissue microarray (TMA) was obtained from Xi׳an Alena Biotechnology Ltd., Co. (Xi’an, Shanxi, China). Association of CMPK protein expression with clinicopathological features of patients with EOC was analyzed after immunohistochemistry staining.

### 2.2 Cell culture, treatment with TGF-β and transduction

OVCAR-3 and SK-OV-3 cells (ATCC, Manassas, VA, USA) were cultured in RPMI-1640 and DMEM (Corning Inc., Manassas, VA, USA), respectively. The cells were treated with 10 ng/ml of recombinant human TGF-β1 (R&D Systems, Minneapolis, MN, USA) for 24 hours. Small interfering RNA (siRNA) was purchased from GenePharma Company (Shanghai, China). The cells were transiently transfected with siRNA mixture (Roche Applied Science, Indianapolis, IN, USA) for 5 h. CMPK-shRNA was constructed with double-strand oligonucleotides. The efficiency of CMPK-shRNA lentiviral transduction was examined by fluorescence microscopy as the construct contains green fluorescent protein (GFP). Knockdown of CMPK was confirmed by qRT-PCR and Western blot.

### 2.3 Transfection of miRNA mimics

Nine miRNA mimics and negative control miRNA were purchased from Guangzhou RiboBio Co., Ltd. (Guangzhou, Guangdong, China). The cells were transfected with miRNA mimics for 5 h and then incubated in a complete medium for up to 72 hours.

### 2.4 Western blot

Protein was detected by Western blot using antibodies specific to phospho-Smad2 and Smad2 (Cell Signalling Technology, Inc. Danvers, MA, USA), active caspase-3 and full sized caspase-3 (Abways Technology, Inc. Shanghai, China).

### 2.5 Flow cytometry

Apoptotic cells were detected by staining cells with APC Annexin-V and propidium iodide (PI) using an Annexin-V Apoptosis Detection Kit (BD Pharminggen, San Diego, CA, USA). Lentivirus infected EOCs were seeded in a 6-well plate and were incubated for 24 hours. Both supernatant and attached cells were collected and resuspended in 100 µl of 1X binding buffer. After adding 5 µl of Annexin-V and/or 5 µl of PI, the cells were incubated in the dark at room temperature for 15 min. After adding 400 µl of 1X binding buffer, the cell population was analyzed by flow cytometry.
